# Serum Selenium, Iron, Zinc, and Copper Concentrations in Renal Transplant Recipients Treated with Mycophenolate Mofetil

**DOI:** 10.1007/s12011-020-02074-2

**Published:** 2020-02-18

**Authors:** Aleksandra Wilk, Dagmara Szypulska-Koziarska, Małgorzata Marchelek-Myśliwiec, Wojciech Głazek, Barbara Wiszniewska

**Affiliations:** 1 Department of Histology and Embryology, Pomeranian Medical University in Szczecin, Powst. Wlkp. 72, 70-111 Szczecin, Poland; 2 Department of Nephrology, Transplantology and Internal Medicine, Pomeranian Medical University, Powst. Wlkp. 72, 70-111 Szczecin, Poland

**Keywords:** Mycophenolate mofetil, Renal transplant recipients, Bioelement

## Abstract

There are data available in the literature on bioelement concentrations in the serum of various groups of patients; however, very little is known about the serum concentration of selenium (Se), iron (Fe), zinc (Zn), and copper (Cu) in renal transplant patients treated with immunosuppressive drugs, including mycophenolate mofetil (MMF). Monitoring of serum bioelement concentrations in renal transplant recipients is of profound importance, as the proper bioelement levels seem to prolong the normal function of the transplanted organ. Thus, the aim of this current study was to examine and carry out comparative analysis involving serum concentrations of Se, Fe, Cu, and Zn of renal transplant recipients treated with MMF and without MMF. The material consisted of blood samples from 115 patients of the Department of Nephrology, Transplantology, and Internal Medicine of Independent Public Clinical Hospital No. 2, Pomeranian Medical University, in the city of Szczecin in the northwestern Poland. Serum Se, Fe, Cu, and Zn levels were quantified by inductively coupled mass spectroscopy (ICP-MS). Taking into account all patients, MMF increases Cu level. Cu and Fe concentrations were significantly higher in women treated with MMF; in group of younger patients treated with MMF, Se level was significantly lower comparing with those whose regimen did not include MMF. Additionally, MMF in combination with prednisone increased Se concentration in blood of transplant recipients. Our study highlights that trace elements should be monitored to allow for an early detection of trace elements deficits, which can easily be corrected for by an adjusted diet or supplemental intake.

## Introduction

Renal transplant recipients need to have their biochemical parameters monitored regularly, including creatinine level, hepatic enzyme concentrations, and blood ion levels. Monitoring serum bioelement concentrations in renal transplant recipients is of profound importance, as the proper bioelement levels seem to prolong the normal function of the transplanted organ [16, 30]. Iron (Fe) status has not been thoroughly studied in renal transplant recipients; however, according to Malyszko et al. [22], Fe deficiencies were detected in 62.4% of long-term kidney recipients without anemia [22]. Hemodialysis patients are often supplemented with Fe to guarantee iron stores for erythropoiesis and to maintain hemoglobin targets. The second element, zinc (Zn), is necessary for proper functioning of organism. Its role is extensive, and includes the activation of many enzymes. Together with copper (Cu), it participates in antioxidative stress mechanisms [9]. Zinc protects cells, tissues, and organs (including the kidneys) against free radicals by activating Cu/Zn superoxide dismutase and reducing the production of reactive oxygen species [1, 9, 24]. Another bioelement, selenium (Se), also affects redox status on account of its antioxidative properties [8, 23]. Notably, it can be used as a cancer marker [20, 21]. According to the statistics, transplant recipients are high-risk cancer patients [4, 5, 37]. Patients on dialysis and transplant recipients experienced over 2.5 times greater increased risk of death from cancer than the general population [4]. Moreover, the incidence of urothelial carcinoma has been proven to be significantly higher in renal transplant recipients than among the general population [37]. The long-term study suggested that transplant recipients exhibited increased risk of being diagnosed with a more advanced tumor than the general population [5]. The aforementioned highlights that trace elements should be monitored to allow for an early detection of trace elements deficits, which can easily be corrected for by an adjusted diet or supplemental intake.

Transplant recipients need to use immunosuppressive drugs for their antirejection properties. The selection of immunosuppressive drug in the post-transplant therapy stage depends on many factors, including the etiology of the underlying disease and biochemical blood parameters, which embrace creatinine levels, and the coexistence of viral infection (CMV, BKV, HCV, HBV). Immunosuppressive therapy is rarely monotherapy; the regimen generally involves multiple drug protocols, including calcineurin inhibitors (CNIs), mTOR inhibitors, inhibitors of cell division, and glucocorticoids. One of the most widely applied immunosuppressive drugs is the mycophenolic acid (MPA) derivative—mycophenolate mofetil (MMF). This is an immune-modulatory drug that inhibits inosine monophosphate dehydrogenase (IMPDH). It inhibits the proliferation and clonal expansion of T and B lymphocytes while preventing the production of cytotoxic T cells [10, 13, 25]. Mycophenolate mofetil is mostly used with calcineurin inhibitors to prolong allograft function, partly due to its antioxidant properties [11]. Mycophenolic acid is derived from several species of *Penicillium* and shows potent bioactivity [29].

Numerous studies have examined the effect of exogenous factors—including smoking, diet, and amalgam—on heavy metal, macroelement, and microelement concentrations in various organs and tissues, including blood [3, 14, 32, 35]. However, little is still known about the effect of various medicines, including immunosuppressive drugs, on the blood levels of these chemical elements. It has been shown that an immunosuppressive regimen based on MMF, which increases the concentration of Cu and Zn in the blood serum, also reduces the concentration of Na [16]. In addition, MMF disturbs Fe levels in the blood [16]. Plasma iron concentrations were significantly higher in patients treated with MMF [28]. This emphasizes the need for increased awareness among clinicians to ensure the proper immunosuppressive drug regimen, taking into account the role of drugs in altering serum bioelement level. Our previous study indicated that cadmium, lead, and mercury concentrations are statistically lower in renal grafts than in cancerous kidneys [32]. Moreover, we documented decreased levels of vanadium and copper in renal grafts [33, 35]. This may have been caused by the immunosuppressive drugs used by the transplant recipients. The aim of this current study was thus to examine and compare the serum concentrations of selenium, iron, copper, and zinc of renal transplant recipients treated with MMF and without MMF.

## Material and Methods

The study was approved by the Bioethics Committee of the Pomeranian Medical University (decision KB-0012/74/17). The current research has been carried out in accordance with The Code of Ethics of the World Medical Association (Declaration of Helsinki) for experiments involving humans.

The research was carried out from 2017 to 2019. The material consisted of blood samples from 115 patients of the Department of Nephrology, Transplantology, and Internal Medicine of Independent Public Clinical Hospital No. 2, Pomeranian Medical University, in the city of Szczecin in northwestern Poland. The material was obtained from patients who had undergone renal transplantation and in whom the function of the graft was stable for over 6 months. Patients were randomly selected. All patients obtained written information describing the prospective research, including the purposes of the study and the approval form for their participation. A 10-cm^3^ blood sample was obtained during the diagnostic workup and was collected into tubes certified for quantification of trace metals (Vacutainer System, royal blue cap). Blood samples were centrifuged within 30 and 120 min of collection to separate the serum from the cellular fraction. The serum samples were stored at − 80 °C until the selenium, iron, zinc, and copper assays were performed. The blood samples were obtained from women aged from 24 to 71 years and from men aged from 28 to 72 years, since these were patients of the Department of Nephrology, Transplantology and Internal Medicine Independent Public Clinical Hospital No. 2, Pomeranian Medical University, who had undergone renal transplantation. The patients were divided into two main groups: (i) MMF+, patients using regimen including mycophenolate mofetil, and (ii) a control group (MMF−) of patients without MMF intake. We also divided the patients in two groups: those over and those under 50 years of age, since there are metabolic differences. The number of patients in group is unequal; thus, immunosuppressive therapy is chosen individually and depends on etiology of the main disease. Additionally, our analysis took into consideration gender and codrug intake. Some renal transplant recipients receive combination of two drugs, and some of them are treated with protocol based on three drugs; therefore, we analyzed the results in two aspects, depending on number and type of drugs included in regimen. We analyzed bioelement concentrations in patients in terms of calcineurin inhibitor intake. We took into account MMF intake (MMF+ or MMF−) and cyclosporine (CsA) or tacrolimus (Tac) intake. Furthermore, we analyzed potential differences between tested elements taking into account three drugs in regimen: MMF + type of calcineurin inhibitor + Pred. Serum creatinine level, GFR, and ALT were obtained as a routine control examination (Table 1).

**Table 1:** Characteristics of the test group (MMF+: treatment with mycophenolate mofetil) and the control group (MMF−: regimen without mycophenolate mofetil)

	MMF+ (*n* = 90)	MMF− (*n* = 25)	All patients (*n* = 115)
Age (years, AM ± SD)	52.12 ± 12.97	47.96 ± 13.71	51.2 ± 13.1
Creatinine level (mg/dl, AM ± SD)	1.51 ± 0.64	1.94 ± 1.41	1.61 ± 0.88
GFR (ml/min/m^3^, AM ± SD)	55.00 ± 22.01	48.76 ± 23.45	53.63 ± 22.41
ALT (IU/L, AM ± SD)	22.58 ± 14.21	22.45 ± 10.75	22.55 ± 13.51
Females/males (*n*)	40/50	14/11	54/61

### Element Assay

Serum selenium, iron, zinc, and copper levels were quantified by inductively coupled mass spectroscopy (ICP-MS NexION 350D, PerkinElmer) using methane to reduce polyatomic interferences, following Lubinski et al. [20, 21]. Calibration standards were prepared by diluting 10 mg/L Multi-Element Calibration Standard 3 (PerkinElmer Pure Plus, PerkinElmer Life and Analytical Sciences, USA) with a blank reagent consisting of a 0.65% solution of nitric acid (Merck, Germany) and 0.002% Tryton X-100 (PerkinElmer, USA). Calibration curves were created using four different concentrations: 0.1 μg/L, 0.5 μg/L, 1 μg/L, and 2 μg/L. Germanium (PerkinElmer Pure, PerkinElmer Life and Analytical Sciences, USA) was used as an internal standard, and ClinChek Plasma Control Level I (Recipe, Germany) was used as a reference material, measured after each of the six samples: where the difference of the reference material measurements exceeded 5%, the entire series was repeated. Each sample was measured in duplicates in different analytical runs. Prior to analysis, all samples were centrifuged (6000 rpm, 15 min) and the supernatant was diluted 100 times with the reagent blank. Technical details, plasma operating settings, and mass spectrometer acquisition parameters are available on request.

### Statistical Analysis

The statistical analysis employed StatSoft Statistica 13.3 software and Microsoft Excel 2018. To evaluate the compliance of the results with the expected normal distribution, Kolmogorov–Smirnov (KS) tests with the Lilliefors correction were used (*p* < 0.05). Median (Med) and percent coefficient of variation (CV) were established for the concentrations of Zn, Cu, Fe, and Se. In addition, the medians of the bioelements in serum were compared and contrasted between different patients groups. As the data distribution was not consistent with the expected normal distribution, the Mann–Whitney *U* test was used (MWU; *p* < 0.05). Spearman’s rank correlation coefficients (*r*_s_) were also determined.

## Results

The 115 renal transplant recipients are characterized in Table 1.

### Serum Elements Level in All Patients

Both the KS test and the KS test with the Lilliefors correction failed to show characteristics of normal distribution. Therefore, the mean concentrations of the elements were compared using the nonparametric MWU test. The bioelement concentrations can be arranged in the ascending series Se < Zn < Fe < Cu (Table 2). Comparing the concentrations of elements across all patients, higher levels of Se and Zn were noted in the control group (MMF−) than in MMF+; these concentrations amounted to 73.64 μg/L vs. 67.99 μg/L and 695.49 μg/L vs. 690.63 μg/L, respectively. Patients treated with MMF displayed higher levels of both Fe and Cu than did patients who were not treated with MMF, at 876.77 μg/L vs. 750.64 μg/L and 939.03 μg/L vs. 862.29 μg/L, respectively. Additionally, the difference was statistically significant in the case of Cu (*p* = 0.03; Table 2).

**Table 2:** Bioelement concentrations in serum of renal transplant recipients (MMF+: treatment with mycophenolate mofetil; MMF−: regimen without mycophenolate mofetil; CV: coefficient of variation in %; *p < 0.05, statistically significant difference)

Parameter	Se (μg/L)	Zn (μg/L)	Fe (μg/L)	Cu (μg/L)
	All patients			
	(*n* = 115)			
Median	69.15	692.30	849.32	921.86
Min–max	36.59–109.89	398.38–3615.26	193.00–2445.23	576.21–1658.95
CV	20.25	47.39	41.52	22.36
	MMF+			
	(*n* = 90)			
Median	67.99	690.63	876.77	939.03*
Min–max	36.59–108.63	402.51–3615.26	193.00–2445.23	576.21–1658.95
CV	19.84	50.52	41.14	23.11
	MMF−			
	(*n* = 25)			
Median	73.64	695.49	750.64	862.29
Min–max	45.24–109.82	398.38–1748.09	363.13–1884.28	612.72–1234.13
CV	21.06	33.81	42.55	15.27
	Females, MMF+			
	(*n* = 40)			
Median	65.36	686.07	867.95*	1037.66*
Min–max	40.41–108.64	439.98–3615.26	193.00–2445.23	697.73–1658.96
CV	20.31	63.54	45.88	21.45
	Females, MMF−			
	(*n* = 14)			
Median	73.72	680.41	652.04	855.42
Min–max	45.24–104.87	476.33–1012.05	363.13–1192.46	612.72–1234.13
CV	19.46	20.14	34.79	18.33
	Males, MMF+			
	(*n* = 50)			
Median	70.42	701.15	906.01	870.04
Min–max	36.59–96.56	402.50–2262.06	396.99–1863	576.21–1380.26
CV	19.66	35.52	37.11	21.53
	Males, MMF−			
	(*n* = 11)			
Median	71.63	711.01	903.42	869.91
Min–max	54.31–109.82	398.38–1748	377.62–1884.28	729.42–999.57
CV	23.21	42.76	39.71	10.81
	< 50, MMF+ (*n* = 32)			
Median	66.09*	694.81	842.79	909.18
Min–max	40.40–92.41	510.26–986.02	193.01–1615.35	576.21–1658.95
CV	18.09	15.72	38.56	25.03
	< 50, MMF− (*n* = 11)			
Median	76.01	774.24	682.34	842.84
Min–max	61.81–109.82	398.38–1748.09	404.57–1884.28	612.72–1052.34
CV	18.74	45.92	53.62	15.01
	> 50, MMF+ (*n* = 58)			
Median	69.13	687.25	958.82	975.29
Min–max	36.59–108.63	402.51–3615.26	396.99–2445.23	578.03–1618.11
CV	20.86	59.76	41.91	22.16
	> 50, MMF− (*n* = 14)			
Median	70.95	693.89	933.99	920.64
Min–max	45.24–108.21	519.77–894.54	363.13–1312.08	729.42–1234.13
CV	21.87	13.21	34.72	14.99

### Impact of MMF Intake on Bioelement Levels in Serum, by Sex

We further divided all patients by sex and considered the effect of MMF intake on bioelement levels in serum in these groups, detecting some significant differences. Women who used MMF had significantly higher concentrations of Fe and Cu than women who were not treated with MMF, with values of 867.95 and 1037.66 μg/L (*p* = 0.02) vs. 652.04 μg/L and 855.42 μg/L (*p* = 0.005; Table 2). Serum bioelement levels in males showed no statistically significant differences due to MMF intake (Table 2).

### Effect of MMF Intake on Bioelement Levels, by Age

In the younger group of patients, Se concentration significantly decreased in the blood of MMF+ patients compared with MMF− patients, with levels of 66.09 μg/L vs. 76.01 μg/L, respectively (Table 2). No significant differences were observed in patients over 50 (Table 2).

### Bioelement Concentrations and Cotherapy

#### Two Drugs Based Therapy

**Fig. 1:**
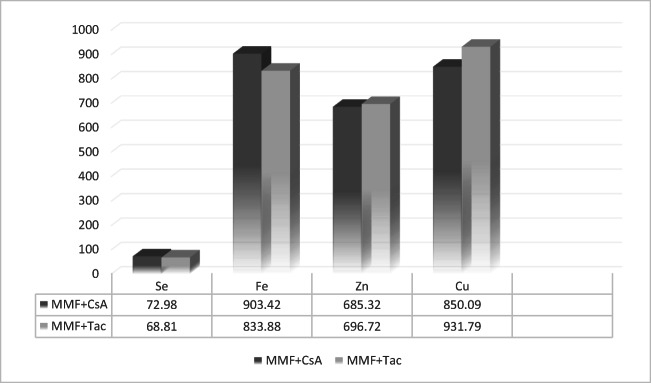
Bioelement concentrations in micrograms per liter in MMF+ patients, by intake of calcineurin inhibitor as a codrug (MMF + CsA: treatment with mycophenolate mofetil and cyclosporine A; MMF + Tac: treatment with mycophenolate mofetil and tacrolimus)

No statistical differences were observed between the concentration of the elements with calcineurin inhibitor intake; however, analysis showed lower concentrations of Cu in the blood of patients who took CsA with MMF (*n* = 17) rather than tacrolimus with MMF (*n* = 98). Furthermore, we observed that Fe concentration was higher in CsA+ patients than in those taking tacrolimus (Fig. 1).

**Fig. 2:**
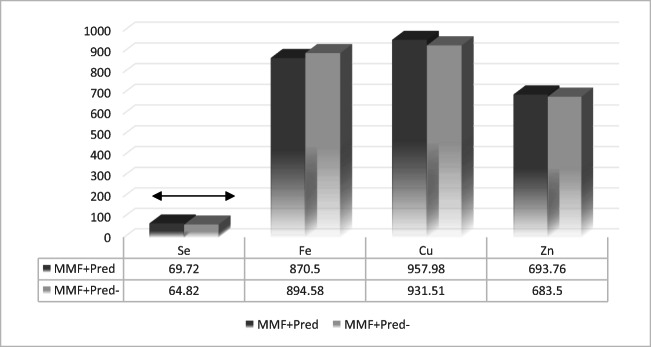
Bioelement concentrations in micrograms per liter in MMF+ patients, by intake of prednisone as a codrug (MMF + Pred: treatment with mycophenolate mofetil and prednisone; MMF + Pred−: treatment with mycophenolate mofetil without prednisone; black arrow: statistically significant difference)

**Fig. 3:**
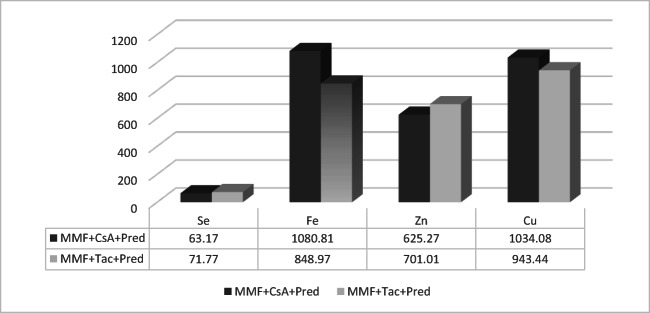
Bioelement concentrations in micrograms per liter in MMF + patients, by intake of calcineurin inhibitor as a codrug in three drugs based regimen (MMF + CsA + Pred: treatment with mycophenolate mofetil and cyclosporine A and prednisone; MMF + Tac + Pred: treatment with mycophenolate mofetil and tacrolimus and prednisone)

These results considered MMF+ patients in terms of their prednisone intake (Fig. 2). As Fig. 3 shows, the Mann–Whitney *U* test results displayed significant difference in selenium levels between patients treated with prednisone (Pred+, *n* = 54) and those not (Pred−, *n* = 36) (*p* = 0.04).

#### Three Drugs Based Therapy

##### Bioelement Levels, by Calcineurin Inhibitor

No statistically significant differences were observed in concentrations of trace elements between patients whose regimen was based on MMF, CsA, and Pred (*n* = 6) versus MMF, Tac, and Pred (*n* = 48) (Fig. 3).

##### Bioelement Levels, by mTOR Inhibitors

Regarding mTORs, as codrugs, the number of patients was not numerous enough to obtain reliable statistical analysis (data not shown).

### Correlations Between Bioelement Concentrations

Furthermore, Spearman’s rank correlation coefficient showed there was a synergistic relationship between Zn and Se both in the control group (MMF−) and in the patients treated with MMF (MMF+; *r*_s_ = 0.49, *p* < 0.05) and additionally in all patients (*r*_s_ = 0.47, *p* < 0.05; Table 3).

**Table 3:** Correlation coefficients of Se, Fe, Zn, and Cu in renal transplant recipients, by MMF intake (MMF+: treatment with mycophenolate mofetil; MMF−, regimen without mycophenolate mofetil)

Correlated elements	Correlation coefficients in		
	MMF+	MMF−	All patients
Se/Fe	0.1	− 0.04	0.04
Se/Zn	0.49*	0.49*	0.47*
Se/Cu	− 0.11	− 0.15	− 0.14
Fe/Zn	0.06	0.09	0.08
Fe/Cu	− 0.09	− 0.01	− 0.06
Zn/Cu	0.14	− 0.25	0.08

*p < 0.05, statistically significant difference

## Discussion

There are data available in the literature on bioelement concentrations in the serum of various groups of patients; however, very little is known about the serum concentration of selenium, iron, zinc, and copper in terms of the intake of immunosuppressive drugs, including mycophenolate mofetil, which is one of the most commonly used. The results of our study of 115 renal transplant recipients from Szczecin, Poland suggest that MMF affects serum bioelements concentrations. Interestingly, the concentrations of Fe and Cu in the serum of patients treated with MMF were higher than in those not treated with MMF. Of note, the difference in Cu levels was statistically significant (*p* = 0.03). Copper is an important trace element that serves as a structural ion in superoxide dismutase (SOD), an antioxidant enzyme that reduces oxidative stress [1]. Our results suggest that MMF evidently increased the level of Cu in blood, which may be associated with the fact that MMF inhibits ROS production by increasing the activity of Cu/Zn SOD [11]. Our findings correlate with those of the study of Kaminska et al. [16], who found Cu levels to be significantly higher in blood serum of renal recipients who used MMF than in patients not using MMF [16]. No other studies of the effect of MMF on serum Cu have been found in the literature, and an explanation of the mechanisms of interaction between MMF and bioelements (including Cu) requires subsequent study.

Multivariate regression analysis has shown the effects of MMF in terms of biological factors (age, sex, and codrug therapy). Taking into account gender, females who were treated with MMF displayed significantly higher Fe and Cu serum levels than females not treated with MMF. As mentioned above, very little is known about the concentration of these elements in terms of the intake of immunosuppressive drugs; however, it has been found that MMF increases Fe and Cu concentrations in the blood [16]. Our data indicate that MMF affects serum Fe levels only in women; this may be associated with MMF metabolism, which probably depends on sex. Additionally, patients with chronic kidney disease and hemodialysis show increased iron losses, reduced iron absorption, and limited iron availability, leading to iron deficiency [36].

We divided the patients into younger and older age groups, taking the age of 50 as the cutoff point. Our results show that patients in the younger group had significantly lower levels of selenium if treated with MMF, as compared to those not receiving MMF. Serum selenium levels in the younger patients were higher in the MMF+ group comparing with MMF− group. Perhaps this difference is associated with other environmental factors, including diet, however it seems that MMF evidently decreases serum Se level in younger patients. Low serum selenium (≤ 70 μg/L) is associated with an increased risk of developing breast cancer [20]. Our results showed Se concentrations below 70 μg/L in younger patients using MMF, which is significant for both patients and clinicians, due to the increased risk of cancer development [20, 21]. Furthermore, a diet rich in micronutrients, including selenium, may help to reduce the overall risk of renal cell carcinoma [6]. There are also data suggesting that selenocysteine could be further developed as novel nutritionally available agents to antagonize cisplatin-induced nephrotoxicity during cancer therapy [19]. Nevertheless, selenium’s anticancer properties remain controversial, and other researchers have rejected the hypothesis [31]. Thus, more research on this bioelement is needed.

There is a number of type of drugs applied in immunosuppressive treatment: (i) inhibitors of the enzyme inosine monophosphate dehydrogenase (IMDH inhibitors), such as mycophenolate mofetil; (ii) calcineurin inhibitors, such as cyclosporine and tacrolimus; (iii) inhibitors the mammalian target of rapamycin (mTORs), such as everolimus and rapamycin; and (iv) corticosteroids, including prednisone [15, 17, 18, 34]. Renal transplant recipients are mostly given multidrug regimens, with one drug from each group. In consequence, the next stage of our study was to determine whether any significant differences are noted in serum bioelement levels for the different types of codrug. The analysis of copper level pointed to lower concentrations of Cu and higher levels of Fe in the blood of patients who used CsA than in patients whose additional drug was tacrolimus. However, this difference was not statistically significant. Interestingly, prednisone significantly increased serum selenium concentration. Our results here are similar to those of other authors. At the end of high-dose prednisone treatment, serum Se level increases simultaneously with enhanced activity of GPX in children [12]. This observation suggests that prednisone affects the serum selenium concentration, which may be associated with the activation of mechanisms against oxidative stress. Taking into account regimens that rely on three drugs no significant differences were observed. The other tested drugs—rapamycin and everolimus belong to mTORs inhibitors. Unfortunately, the number of patients was not numerous enough to obtain the reliable statistical analysis.

Synergistic correlation was observed between Zn and Se concentrations. Both of these elements are associated with redox status [27]. Zinc is a cofactor of Cu/Zn superoxide dismutase, which is suppressed under zinc-deficient conditions. The available data suggest that zinc may also affect the activity of other antioxidant enzymes [27]. Moreover, zinc is correlated with selenium, and so Zn is involved in increasing GPX activity through the modulation of selenium status [2, 27]. There is a scarcity of published data regarding the correlations of various bioelements in the serum of transplant recipients. In the study of Singh et al. [26], Zn was found to have positive correlations with Se in prostate cancer [26], which corresponds with our results.

In all patients included in the study, no deviations were noted from the normal levels of serum bioelements, whereas the study of Kaminska et al. [16] found disturbances through comparisons of selected elements serum concentrations with norms, including decreased levels of sodium [16]. The normal serum concentration is 70–140 μg/L for Se, 0.75–1.0 mg/L for Zn, 0.8–1.2 mg/L for Cu, 55–160 μg/dL for iron in men, and 40–155 μg/dL for iron in women [7, 9].

The limitation of our study was that it was not possible to carry out comparative analysis of the results we obtained with those of other studies, as little is known on the topic; secondly, more patients should be included in the project also to compare the regimens based on mTORs. However, the results obtained are part of a preliminary study. More research in this field clearly needs to be conducted, as transplant recipients are high-risk cancer patients, and it is necessary to monitor their bioelement levels. Our knowledge in this area should be expanded in order to improve the life comfort of transplant recipients and to prolong the proper functioning of transplanted organs.

In summary, the study described here is pioneering and should prove helpful for clinicians in selecting optimal treatment regimen, avoiding the ones that negatively affect the concentrations of bioelements or require dietary supplementation. Our main conclusions are that immunosuppressive regimen based on mycophenolate mofetil affects serum bioelement concentration of renal transplant recipients, in the following way:

1. Increasing Cu level in transplant recipients;
2. Increasing Cu and Fe concentration in women;
3. Decreasing Se level in patients younger than 50 years old;
4. Increasing Se level in combination with prednisone.
